# Down-Grading of Ipsilateral Hydronephrosis by Neoadjuvant Chemotherapy Correlates with Favorable Oncological Outcomes in Patients Undergoing Radical Nephroureterectomy for Ureteral Carcinoma

**DOI:** 10.3390/diagnostics10010010

**Published:** 2019-12-23

**Authors:** Makito Miyake, Nagaaki Marugami, Yuya Fujiwara, Kazumasa Komura, Teruo Inamoto, Haruhito Azuma, Hiroaki Matsumoto, Hideyasu Matsuyama, Kiyohide Fujimoto

**Affiliations:** 1 Department of Urology, Nara Medical University, 840 Shijo-cho, Kashihara, Nara 634-8522, Japan; kiyokun@naramed-u.ac.jp; 2 Department of Radiology, Nara Medical University, 840 Shijo-cho, Kashihara, Nara 634-8522, Japan; maru1225maru@yahoo.co.jp; 3 Department of Urology, Osaka Medical College, 2-7 Daigakumachi, Takatsuki, Osaka 569-8686, Japan; uro072@osaka-med.ac.jp (Y.F.); uro051@osaka-med.ac.jp (K.K.); tinamoto@osaka-med.ac.jp (T.I.); uro004@poh.osaka-med.ac.jp (H.A.); 4 Department of Urology, Graduate School of Medicine, Yamaguchi University, 1-1-1 Minami-Kogushi, Ube 755-8505, Japan; hmatsumo@yamaguchi-u.ac.jp (H.M.); hidde@yamaguchi-u.ac.jp (H.M.)

**Keywords:** ureteral neoplasms, hydronephrosis, neoadjuvant therapy, neoplasm recurrence, survival, disease progression

## Abstract

Few studies have analyzed the details of neoadjuvant chemotherapy (NAC)-induced changes in patients with upper tract urothelial carcinoma. This study aimed to describe the impact of down-grading ipsilateral hydronephrosis by NAC for ureteral carcinoma. An observational study was conducted in 32 patients with cT1-3N0M0 ureteral carcinoma treated with NAC and radical nephroureterectomy. Hydronephrosis was classified into five grades based on computed tomography findings. We focused on the differences between the baseline and post-NAC status of ipsilateral hydronephrosis, radiographic tumor response, and blood markers. Down-grading, no change, and up-grading was observed in 10 (31%), 21 (66%), and 1 (3%) patients, respectively. In univariate analysis, locally advanced disease (cT3), severe hydronephrosis (grade 3/4) at baseline, no change/up-grading of hydronephrosis after NAC, and pathological lymphovascular involvement were identified as potential prognostic factors of progression-free and cancer-specific survival after radical nephroureterectomy. Locally advanced disease (cT3) at baseline and no change/up-grading of hydronephrosis by NAC were independently associated with poor progression-free survival. Notably, none of the patients with NAC-induced down-grading of hydronephrosis died of ureteral carcinoma during the follow-up. We reported the prognostic impact of down-grading of ipsilateral hydronephrosis, which could serve as a useful aid or clinical marker for decision-making.

## 1. Introduction

Upper tract urothelial carcinoma (UTUC) is a rare, heterogeneous, and aggressive malignancy with a poor prognosis [1]. Despite the significant advances made in surgical skill, diagnostic techniques, and identification of clinicopathological and biological prognostic factors, the clinical outcomes of UTUC have not significantly improved over the decades [1,2,3,4]. Recently, the results of a phase III randomized controlled trial of adjuvant chemotherapy (AC) vs. non-AC surveillance in pT2-4 N0-3 M0 UTUC (POUT study) have shown that AC prolonged progression-free survival after radical nephroureterectomy (RNU) [5]. Moreover, the trial was terminated early because the clinical efficacy outcomes favored the AC arm. Gregg et al. reported a systematic review and meta-analysis based on 13 comparative studies, and concluded that perioperative chemotherapy including AC and neoadjuvant chemotherapy (NAC) could provide a survival benefit after RNU [6]. However, the roles of perioperative systemic chemotherapy for UTUC and an optimized treatment sequence remain unclear because of the scarcity of evidence.

There are accumulating data suggesting that patients with high-risk UTUC should be considered for NAC in an attempt to administer a sufficient dose of cisplatin before RNU [7]. To date, NAC has not been widely accepted as a routine treatment strategy for locally advanced UTUC. This seems predominantly because clinicians consider that not all patients benefit from systemic chemotherapy and it can cause toxicities which can delay RNU. Several prognostic factors at baseline have been identified to support decision making in the pursuit of personalized care [8,9,10,11,12,13], including radiographic variables, inflammatory markers, nutritional indices, and molecular markers. Some studies analyzed the relationship of hydronephrosis at baseline to poor outcomes [10,11,12,13]. Cho et al. uniquely reported tumor-caused hydronephrosis grades ranging from one to four, and concluded that there is a positive association between hydronephrosis grade and advanced pathological finding and oncological outcomes [13].

Few studies have analyzed the details of NAC-induced changes such as down-grading of ipsilateral hydronephrosis and shrinkage of the ureteral tumor. In this study, to establish the role of perioperative chemotherapy, we investigated whether a positive response to NAC correlates with better outcomes after RNU.

## 2. Materials and Methods

### 2.1. Study Cohort and Data Collection

This multi-institutional study was approved by the research ethical committee of each participating institute (project code: 1258, date of approval: 20 August 2018). Informed consent was obtained for all human subjects. The study was conducted in compliance with the study’s protocol and following the provisions of the Declaration of Helsinki (2013).

**Figure 1:**
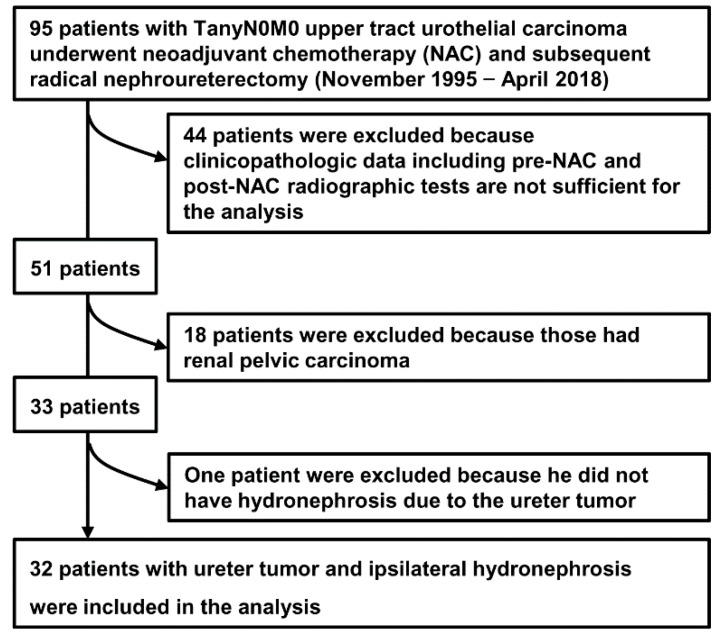
Flowchart for the creation of the cohort data set.

We retrospectively reviewed our cohort of 95 patients with non-metastatic UTUC treated with NAC and subsequent RNU between November 1995 and April 2018. The study cohort was restricted, as follows: (1) cT1-T3N0M0 ureteral carcinoma, (2) presence of tumor-induced ipsilateral hydronephrosis, (3) radiographic urinary tract evaluation of both pre-NAC and post-NAC status, and (4) sufficient clinicopathological data including follow-up information. The restriction left 32 patients (33.6%) who were eligible for the analysis (Figure 1).

The procedure of RNU and the postoperative follow-up protocol have been described in our previous report [1]. The patterns of recurrence sites of UTUC after RNU are diverse (urinary tract, lymph nodes, liver, bone, and lungs). Intravesical recurrence (IVR) after RNU was defined as an intravesical tumor that was pathologically confirmed as urothelial carcinoma. Extra-urinary recurrence, hereinafter referred to as “progression,” was defined as local recurrence or distant metastasis to the lymph nodes, bone, or other visceral organs after RNU. The clinicopathological characteristics of the patients, including age, sex, laboratory data, tumor location, tumor diameter, estimated tumor volume, radiographic data, and pathological findings of the RNU specimen, were all obtained from the medical charts.

### 2.2. Preoperative Systemic Chemotherapy

The planned treatment usually involved 2–3 cycles of NAC and 2–3 cycles of AC with gemicitabine/cisplatin, methotrexate/vinblastine/Adriamycin/cisplatin, or other platinum-based regimens including carboplatin. AC usually started within 3 months after RNU. The detailed dose was described previously [1]. The regimen was selected by the attending clinician mostly based on renal function, comorbidities, and performance status.

### 2.3. Image Interpretation for Ureteral Tumor and Hydronephrosis

All computed tomography (CT) images taken before and after NAC were uploaded in a cloud medical imaging platform (Ambra Health, New York, NY, USA). The images were reevaluated and interpreted by a radiologist (N.M.) with special expertise in urogenital imaging. The investigator was blinded to any other clinicopathological variables. Tumor stage (according to the 2010 American Joint Committee on Cancer tumor-node-metastasis staging system), long diameter of the tumor, estimated tumor volume, and ipsilateral hydronephrosis were determined based on multiplanar reconstruction including axial, sagittal, and coronal CT images. Tumor response was evaluated by the response evaluation criteria in solid tumors (RECIST) v1.1 and estimated tumor volume. Tumor volumes (cm^3^) were estimated using measurements of length × width × height × (π/6) [14]. Response was defined as post-NAC volume/pre-NAC volume × 100 and categorized into four groups, as follows: complete response (CR, 100% remission), partial response (PR, ≥30% remission), stable disease (<30% remission to >20% increase), and progressive disease (≥20% increase). Ipsilateral hydronephrosis was graded as 0–4 according to the previously reported classification [10,13]. We focused on the differences between pre-NAC and post-NAC status in terms of long diameter of the tumor, estimated tumor volume, and ipsilateral hydronephrosis.

### 2.4. Statistical Analysis

Progression-free survival (PFS), cancer-specific survival (CSS), and IVR-free survival were calculated from the date of RNU. Survival rates were analyzed using the Kaplan-Meier method and compared using the log-rank test for univariate analysis. Multivariate analyses were used to identify independent prognostic variables based on a stepwise Cox proportional hazard regression model and variables that potentially affected survival (*p* < 0.05 in univariate analysis). Data were analyzed using the SPSS software (version 21; IBM SPSS, Armonk, NY, USA) and plotted using the PRISM software (version 7.00; GraphPad, San Diego, CA, USA). The statistical significance was set at *p* < 0.05, and all reported *p* values were two-sided.

## 3. Results

### 3.1. Baseline Characteristics Including Ipsilateral Hydronephrosis Grade

**Figure 2:**
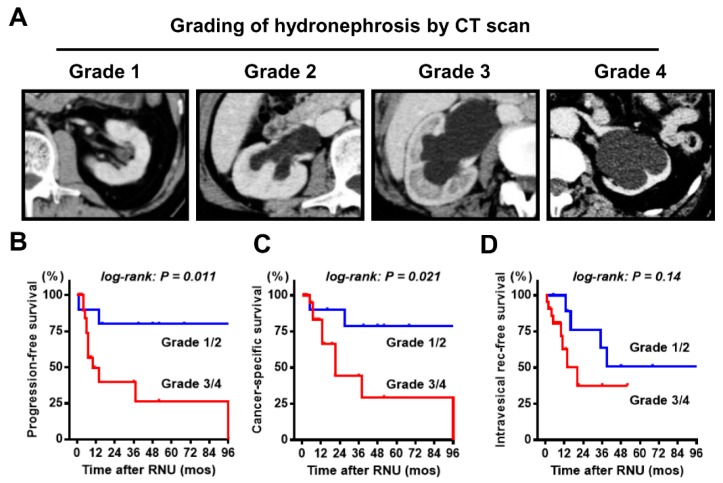
Grading of ipsilateral hydronephrosis and correlation with the outcomes. (**A**) Ipsilateral hydronephrosis is graded as follows: grade 1 = pelvic dilatation only, grade 2 = mild calix dilatation, grade 3 = severe calix dilatation, and grade 4 = calix dilatation accompanied by renal parenchymal atrophy. Curves for progression-free survival (**B**), cancer-specific survival (**C**), and intravesical recurrence-free survival (**D**) are plotted and compared according to hydronephrosis (grade 1/2 vs. grade 3/4). CT = computed tomography, RNU = radical nephroureterectomy.

**Figure 3:**
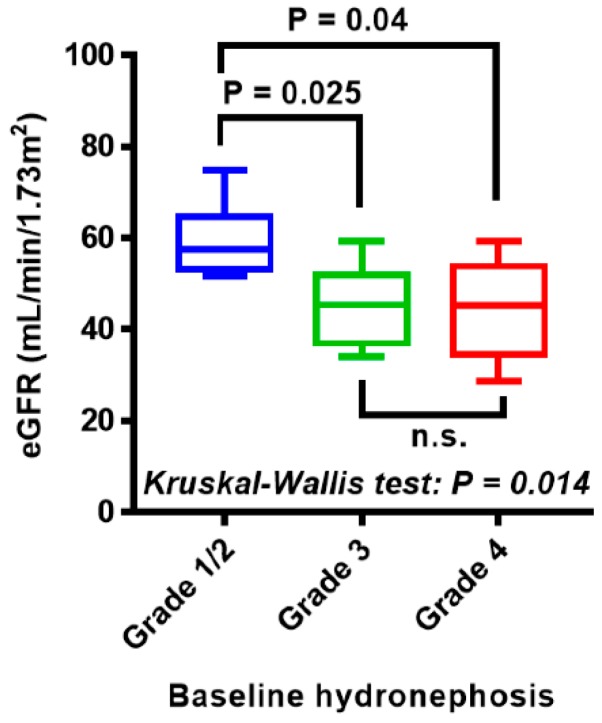
Association between hydronephrosis at baseline and renal function. The estimated. glomerular filtration rate (eGFR) is depicted using Tukey box plots. The horizontal lines withinboxes indicate median levels. *p* values are based on the Kruskal-Wallis test followed by post hoc Dunn’s test.

Table 1 shows the clinicopathological characteristics and treatment options of 32 patients with ureteral carcinoma. More than half of the patients had locally advanced tumor (cT3). Four representative images of grade 1 to 4 hydronephrosis are shown in Figure 2A. Grades 1, 2, 3, and 4 were observed in 2 (6%), 8 (25%), 11 (34%), and 11 (34%) patients at baseline, respectively. The median follow-up period after RNU was 16 months. During the follow-up period, 14 patients (44%) and 12 patients (38%) experienced progression and IVR, respectively, and 11 patients (34%) died of ureter cancer. Further, we analyzed the association between the baseline hydronephrosis grade and oncological outcomes. Univariate analysis for progression, cancer-specific death, and IVR demonstrated that grade 3/4 hydronephrosis was associated with shorter PFS and CSS, but not with IVR-free survival (Figure 2B–D). As expected, the renal function of patients with grade 3/4 hydronephrosis was significantly decreased compared with those with grade 1/2 hydronephrosis (mean ± standard deviation estimated glomerular filtration rate, 44 ± 11 vs. 57 ± 12 mL/min/1.73 m^2^; Figure 3).

### 3.2. Clinical Impact of Change in Hydronephrosis Grade Induced by NAC

**Figure 4:**
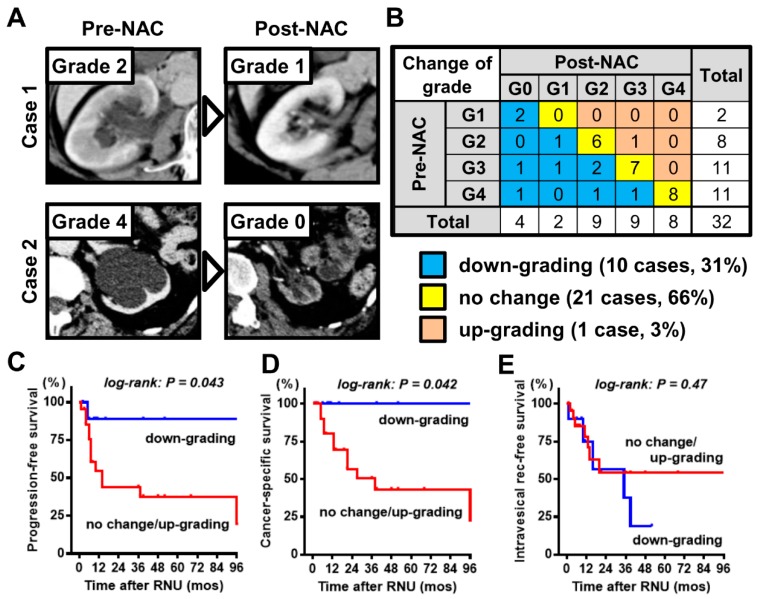
Association between change in hydronephrosis grade induced by NAC and the outcomes. (**A**) Representative images from two patients who experienced down-grading of hydronephrosis by NAC. Case 1: 53-year-old female patient with cT3 right ureteral tumor. Case 2: 67-year-old male patient with cT3 left ureteral tumor. (**B**) Tabulation of the changes in hydronephrosis grade induced by NAC. Curves for progression-free survival (**C**), cancer-specific survival (**D**), and intravesical recurrence-free survival (**E**) are plotted and compared according to the change in hydronephrosis grade induced by NAC (down-grading vs. no change/up-grading). NAC = neoadjuvant chemotherapy, RNU = radical nephroureterectomy.

Post-NAC hydronephrosis was graded and compared with the pre-NAC (baseline) status. Representative images of down-graded hydronephrosis are shown in Figure 4A. Only 1 patient experienced up-grading from grade 2 to 3, whereas dramatic down-grading from grade 4 to 0 was observed in a 67-year-old male patient with cT3 left lower ureteral carcinoma (case 2 in Figure 4A). Of the 32 patients, 10 (31%), 21 (66%), and 1 (3%) showed down-grading, no change, and up-grading, respectively (Figure 4B). The pathological examination of the surgical specimen after NAC showed no cancer cells in the ureter (pT0), and the patient has survived for 12 months without any disease recurrence and progression thus far. Univariate analysis demonstrated that down-grading of hydronephrosis was associated with better PFS and CSS, but not IVR-free survival (Figure 4C–E). Table 2 shows the association between the baseline characteristics and change of hydronephrosis grade after NAC. There was a marginal association between low age and down-grading of hydronephrosis (*p* = 0.09). Moreover, we investigated the association between the number of NAC cycles and change in hydronephrosis grade. Four of nine patients (44%) treated with one cycle experienced down-grading, whereas around 25% of patients treated with two or three cycles experienced down-grading of ipsilateral hydronephrosis. There was no significant association between the number of NAC cycles and change in hydronephrosis grade (Table 3).

### 3.3. Analysis of Tumor Response to NAC

**Figure 5:**
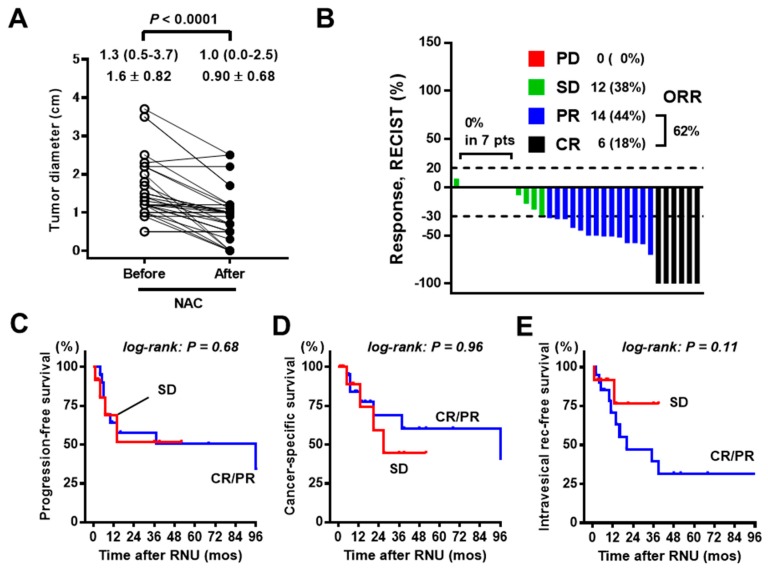
Association between tumor response to NAC by RECIST criteria and the outcomes. (**A**) Dot graphs showing the tumor diameter before and after NAC. Values are shown as median (interquartile range) and mean ± standard deviation. The change was evaluated using the Wilcoxon signed-rank test. (**B**) A waterfall plot depicting tumor response to NAC in 32 patients with ureteral carcinoma. CR = complete response (100% remission), PR = partial response (≥30% remission), SD = stable disease (<30% remission to >20% increase), PD = progressive disease ≥ 20% increase), ORR = objective response rate (CR or PR). Curves for progression-free survival (**C**), cancer-specific survival (**D**), and intravesical recurrence-free survival (**E**) are plotted and compared according to the tumor response to NAC (CR/PR vs. SD). NAC = neoadjuvant chemotherapy, RNU = radical nephroureterectomy.

Figure 5A shows the tumor diameters before and after NAC. The mean response rate was 42% remission, and more than 60% of patients obtained an objective response with respect to the ureteral tumor (Figure 5B; CR in 18% and PR in 44%). There were no patients experiencing PD during NAC in our cohort. In contrast to the results of hydronephrosis, there was no significant association between tumor response and outcomes after RNU (Figure 5C–E). Supplementary Figure S1 shows the analysis of the tumor response to NAC in estimated tumor volume. The results were similar to those of the RECIST criteria.

### 3.4. Prognostic Value of NAC-Induced Changes and Other Potential Factors

Additional analyses were performed to investigate the prognostic values of NAC-induced changes. The pre-NAC and post-NAC mean values of C-reactive protein in blood were 0.45 ± 1.26 and 0.23 ± 0.54 mg/L (*p* = 0.38), respectively, whereas those of the neutrophil-to-lymphocyte ratio were 3.3 ± 2.4 and 3.2 ± 1.9 (*p* = 0.06), respectively. In univariate analysis (Table 4), locally advanced disease (cT3), severe hydronephrosis (grade 3/4) at baseline, no change/up-grading of hydronephrosis after NAC, and pathological lymphovascular involvement in the RNU specimen were identified as potential prognostic factors of PFS and CSS after RNU. However, no factor tested in this study was associated with the IVR risk. Multivariate analysis revealed that locally advanced disease (cT3) at baseline and no change/up-grading of hydronephrosis after NAC were independently associated with poor PFS, whereas hydronephrosis at baseline and pathological lymphovascular involvement were not independent prognostic factors (Table 5). Notably, none of the patients with NAC-induced down-grading of hydronephrosis died of ureteral carcinoma during the follow-up (Figure 4D).

## 4. Discussion

The present study focused on NAC-induced changes in patients with ureteral cancer presenting with hydronephrosis before treatment. To our knowledge, this is the first report to address the association between the down-grading of ipsilateral hydronephrosis and survival outcomes. Previous studies have demonstrated a positive association between baseline hydronephrosis grade and poor outcomes [10,11,13]. The largest-scale study thus far with 401 patients with UTUC failed to show an association between preoperative hydronephrosis and survival [12]. Among the previous studies, only that by Cho et al. excluded patients with renal pelvic carcinoma from the analysis. It seems better to restrict the study cohort to only patients with ureteral carcinoma for analyzing tumor-induced hydronephrosis, because hydronephrosis could not be accurately evaluated and graded in patients with renal pelvic carcinoma. Asai et al. proposed a unique classification of hydronephrosis based on the renal excretion of 18F-flurodeoxyglucose (FDG) on FDG-positron emission tomography/CT [15]. Their results showed that hydronephrosis without FDG excretion, defined as “type 2 hydronephrosis,” was associated with less decline in renal function after RNU and a higher risk of muscle-invasive disease (≥pT2). Therefore, hydronephrosis in UTUC, especially ureteral carcinoma, is believed to be informative and helpful for deciding the treatment strategy.

The potential benefit of NAC for UTUC is originally supported by the preceding clinical trials involving patients with muscle-invasive bladder cancer (MIBC), which provided robust evidence showing improved survival in patients who had NAC [16,17]. Poten et al. compared patients with high-risk UTUC who underwent NAC treatment followed by RNU and those who did not have NAC (matched historical cohort) to conclude that the NAC cohort had better survival in terms of five-year CSS (90% vs. 57%, *p* = 0.0015) [7]. Although there have been accumulating data showing good outcomes of NAC for UTUC, prospective randomized trials are needed to strongly recommend this treatment modality. Moreover, clinicians raised another clinical question about whether there are patients with the highest-risk non-metastatic UTUC requiring both NAC and AC. However, refined patient selection is needed for this highly invasive and long-standing treatment. Millikan et al. conducted a phase III trial evaluating the clinical potential of integrated therapy with radical cystectomy plus five cycles of M-VAC for high-risk MIBC [18]. Although the authors failed to find a preferred sequence (two cycles of NAC plus three cycles of AC vs. five cycles of AC), it is possible to select appropriate patients for such integrated therapy based on clinicopathological characteristics.

NAC consisting of multiagent regimens can cause substantial changes both in the primary tumor and the systemic condition of the patient. Some of these changes may serve as predictive markers for oncological outcomes. In a comprehensive review, Aziz et al. highlighted four retrospective studies evaluating the benefit of NAC for UTUC [19]. Of four studies, two [20,21] reported that the rates of NAC-induced pathological response were 13%–14% CR and 32%–40% PR. In our cohort, pathological CR (pT0) was observed similarly in four patients (13%). Two of four patients died of ureteral cancer during the follow-up. Neither pathological response nor radiographical response was a favorable prognostic factor in the present study. Among the tested NAC-induced changes, only down-grading of hydronephrosis was associated with better clinical outcomes (Table 4). It seems that down-grading of hydronephrosis requires a reduction in ureteral obstruction and the recovery of healthy peristaltic ureteral movement. Both observations may be crucial to achieving better survival benefit after RNU. Our findings suggested that the change in hydronephrosis grade induced by NAC for ureteral carcinoma would be a useful marker for selecting patients who need AC after RNU. To date, the optimal number of cycles of NAC for UTUC has not been determined. Down-grading of hydronephrosis may serve as a good indicator of the optimal number of cycles for each patient.

The present study has several limitations. First, it has a retrospective nature with potential selection bias. For example, some patients were excluded because of missing data. Second, a relatively small number of patients with UTUC-non-MIBC were enrolled in this analysis. Although UTUC is a rare tumor, our collaborative group has a database including more than 1500 patients. However, only a small percentage of patients with ureteral carcinoma underwent NAC treatment before RNU. This would be the common situation worldwide [1,22]. In addition, the studied cohort was from multiple institutions, which could introduce inconsistencies in surgical skills, selection of chemotherapy regimens, follow-up durations, clinical interpretations, and pathological diagnosis.

## 5. Conclusions

Some patients with high-risk UTUC should be managed using integrated multidisciplinary treatments including NAC, extirpative surgery, subsequent AC, immunotherapy, and radiotherapy. We believe that our findings could support clinical decision making. Further, large-scale retrospective observational studies and subsequent prospective trials are needed to better define the real clinical value of down-grading of ipsilateral hydronephrosis in terms of pathological and survival outcomes.

## Figures and Tables

**Table 1:** The characteristics of patients with ureteral carcinoma undergoing neoadjuvant chemotherapy and radical nephroureterectomy.

Variables		*n* (%)
Total		32 (100%)
Age at diagnosis	Mean ± SD	69 ± 8.9
Sex	Male	22 (69%)
	Female	10 (31%)
Location of main tumor	Upper	6 (19%)
	Middle	10 (31%)
	Lower	16 (50%)
Baseline clinical T category ^†^	cT1/2	13 (40%)
	cT3	19 (60%)
Long diameter of index tumor (cm)	Median (IQR)	1.3 (1.0–2.2)
	Mean ± SD	1.6 ± 0.82
Estimated volume of index tumor (cm^3^)	Median (IQR)	0.79 (0.49–2.6)
	Mean ± SD	2.5 ± 4.0
Baseline CRP level	Mean ± SD	0.45 ± 1.26
Baseline NLR	Mean ± SD	3.26 ± 2.40
Hydronephrosis due to ureteral tumor	Grade 1	2 (6%)
	Grade 2	8 (25%)
	Grade 3	11 (34%)
	Grade 4	11 (34%)
Neoadjuvant chemotherapy regimen	GC	24 (75%)
	M-VAC	3 (9%)
	Other	5 (16%)
Pathological T category at RNU ^†^	pT0	4 (13%)
	pTa	2 (6%)
	pT1	6 (19%)
	pT2	8 (25%)
	pT3	11 (34%)
	pT4	1 (3%)
Pathological N category at RNU ^†^	N0	28 (89%)
	N1–2	4 (11%)
Adjuvant chemotherapy	No	28 (89%)
	GC	3 (9%)
	M-VAC	1 (3%)

SD = standard deviation; IQR = interquartile range; RNU = radical nephroureterectomy; CRP = c-reactive protein; NLR = neutrophil-to-lymphocyte ratio; GC = Gemcitabine and Cisplatin; M-VAC = Methotrexate, Vinblastine, Doxorubicin, and Cisplatin; ^†^ the 7th edition of the UICC-AJCC TNM staging system.

**Table 2:** Association between the baseline characteristics and change of hydronephrosis grade after neoadjuvant chemotherapy.

Variables		Change in Hydronephrosis Grade		*p* Value
		Down-Grading	No Change/Up-Grading	
Total		10 (31%)	22 (69%)	-
Age at diagnosis	Mean ± SD	65.1 ± 10.7	71.1 ± 7.5	0.09
Sex	Male	8 (80%)	14 (64%)	0.44
	Female	2 (20%)	8 (36%)	
Location of main tumor	Upper	2 (20%)	4 (18%)	0.64
	Middle	2 (20%)	8 (36%)	
	Lower	6 (60%)	10 (46%)	
Baseline clinical T category ^†^	cT1/2	3 (30%)	7 (32%)	0.92
	cT3	7 (70%)	15 (68%)	
Long tumor diameter (cm)	Mean ± SD	1.9 ± 1.1	1.5 ± 0.68	0.60
Estimated tumor volume (cm^3^)	Mean ± SD	3.7 ± 5.4	2.0 ± 3.4	0.59
Baseline CRP level	Mean ± SD	0.79 ± 2.11	0.30 ± 0.61	0.96
Baseline NLR	Mean ± SD	3.12 ± 1.39	3.33 ± 2.92	0.57
Baseline hydronephrosis	Grade 1	2 (20%)	0 (0%)	0.12
	Grade 2	1 (10%)	7 (32%)	
	Grade 3	4 (40%)	7 (32%)	
	Grade 4	3 (30%)	8 (36%)	
NAC regimen	GC	6 (60%)	18 (82%)	0.33 ^‡^
	M-VAC	0 (0%)	3 (14%)	
	Other	4 (40%)	1 (4%)	

SD = standard deviation; CRP = c-reactive protein; NLR = neutrophil-to-lymphocyte ratio; GC = Gemcitabine and Cisplatin; M-VAC = Methotrexate, Vinblastine, Doxorubicin, and Cisplatin; ^†^ the 7th edition of the UICC-AJCC TNM staging system, ^‡^ Comparison between GC and M-VAC (excluding other).

**Table 3:** Association between the number of neoadjuvant chemotherapy cycles and changes in hydronephrosis grade.

Number of Cycles	Change in Hydronephrosis			Total
	Down-Grading	No Change	Up-Grading	
1	4 (44%)	5 (56%)	0 (0%)	9 (100%)
2	5 (26%)	13 (68%)	1 (5%)	19 (100%)
3	1 (25%)	3 (75%)	0 (0%)	4 (100%)
Total	10 (31%)	21 (66%)	1 (3%)	32 (100%)

**Table 4:** Univariate analysis of baseline and post-NAC variables for progression-free survival, cancer-specific survival, and IVR-free survival in patients with ureteral carcinoma.

Variables	Progression-Free Survival			Cancer-Specific Survival			IVR-Free Survival		
	HR	95% CI	*p*	HR	95% CI	*p*	HR	95% CI	*p*
Age									
70 or less	1			1			1		
more than 70	1.52	0.51–4.5	0.45	1.47	0.43–5.0	0.54	1.07	0.34–3.37	0.91
Sex									
Male	1			1			1		
Female	0.68	0.24–2.0	0.45	0.53	0.16–1.7	0.26	0.65	0.21–2.02	0.46
Baseline clinical T									
cT1/2	1			1			1		
T3	3.53	1.23–10.1	0.03	2.67	0.82–8.70	0.12	1.27	0.41–4.00	0.68
Baseline hydronephrosis									
Grade 1/2	1			1			1		
Grade 3/4	5.10	1.78–14.6	0.01	4.56	1.40–14.9	0.024	2.32	0.75–8.25	0.14
Baseline blood CRP level (mg/dL)									
less than 0.1	1			1			1		
0.1 or more	1.74	0.58–5.18	0.29	1.79	0.52–6.13	0.35	1.33	0.41–4.22	0.13
Baseline NLR									
less than 2.6	1			1			1		
2.6 or more	0.61	0.20–1.90	0.32	0.97	0.27–3.46	0.96	1.31	0.37–4.59	0.59
Change in hydronephrosis									
Down-grading	1			1			1		
No change/up-grading	6.07	1.97–18.7	0.04	4.16	1.07–16.4	0.042	0.66	0.19–2.16	0.47
Response, RECIST criteria									
CR/PR	1			1			1		
SD	1.11	0.32–3.8	0.87	1.5	0.39–6.0	0.69	0.50	0.15–1.7	0.27
Response in tumor volume									
CR/PR	1			1			1		
SD/PD	0.78	0.23–2.6	0.68	0.97	0.26–3.6	0.96	0.22	0.06–1.24	0.11
Response in CRP level									
Decreased	1			1			1		
No change/increased	0.46	0.15–1.70	0.15	1.01	0.22–4.75	0.99	0.24	0.05–1.17	0.09
Response in NLR									
Decreased	1			1			1		
No change/increased	1.06	0.34–3.30	0.91	0.88	0.25–3.10	0.84	0.81	0.23–2.80	0.73
Pathological T at RNU specimen									
pT0	1			1			1		
pTa/1	NA	NA	0.05	NA	NA	0.056	2.32	0.38–14.3	0.44
pT2–4	1.47	0.40–5.52	0.58	1.36	0.34–5.52	0.67	2.98	0.65–13.5	0.27
LVI at RNU specimen									
No	1			1			1		
Yes	4.14	1.10–15.6	0.03	4.10	1.76–47.1	0.0084	2.21	0.41–11.8	0.19

NAC, neoadjuvant chemotherapy; IVR, intravesical recurrence: HR, hazard ratio: CI, confidence interval: CR, complete reseponse: PR, partial response: SD, stable disease: PD, progressive disease; CRP, c-reactive protein; NLR, neutrophil-to-lymphocyte ratio; RECIST, response evaluation criteria in solid tumours; RNU, radical nephroureterectomy: NA, not available: LVI, lymphovascular involvement.

**Table 5:** Multivariate analysis of baseline and post-NAC variables for progression-free survival in patients with ureteral carcinoma.

Variables	Progression-Free Survival		
	HR	95% CI	*p* Value
Baseline clinical T			
cT1/2	1		
T3	6.12	1.45–25.8	0.014
Change in hydronephrosis			
Down-grading	1		
No change/up-grading	10.4	1.25–86.4	0.030

## Supplementary Materials

The following are available online at https://www.mdpi.com/2075-4418/10/1/10/s1, Figure S1: Association between response to neoadjuvant chemotherapy in tumor volume and the outcomes.
