# Supplementary figures and images for: Down-Grading of Ipsilateral Hydronephrosis by Neoadjuvant Chemotherapy Correlates with Favorable Oncological Outcomes in Patients Undergoing Radical Nephroureterectomy for Ureteral Carcinoma
Source: Diagnostics (Basel). 2019 Dec 23;10(1):10. doi: 10.3390/diagnostics10010010 (PMC7168216; doi:10.3390/diagnostics10010010)

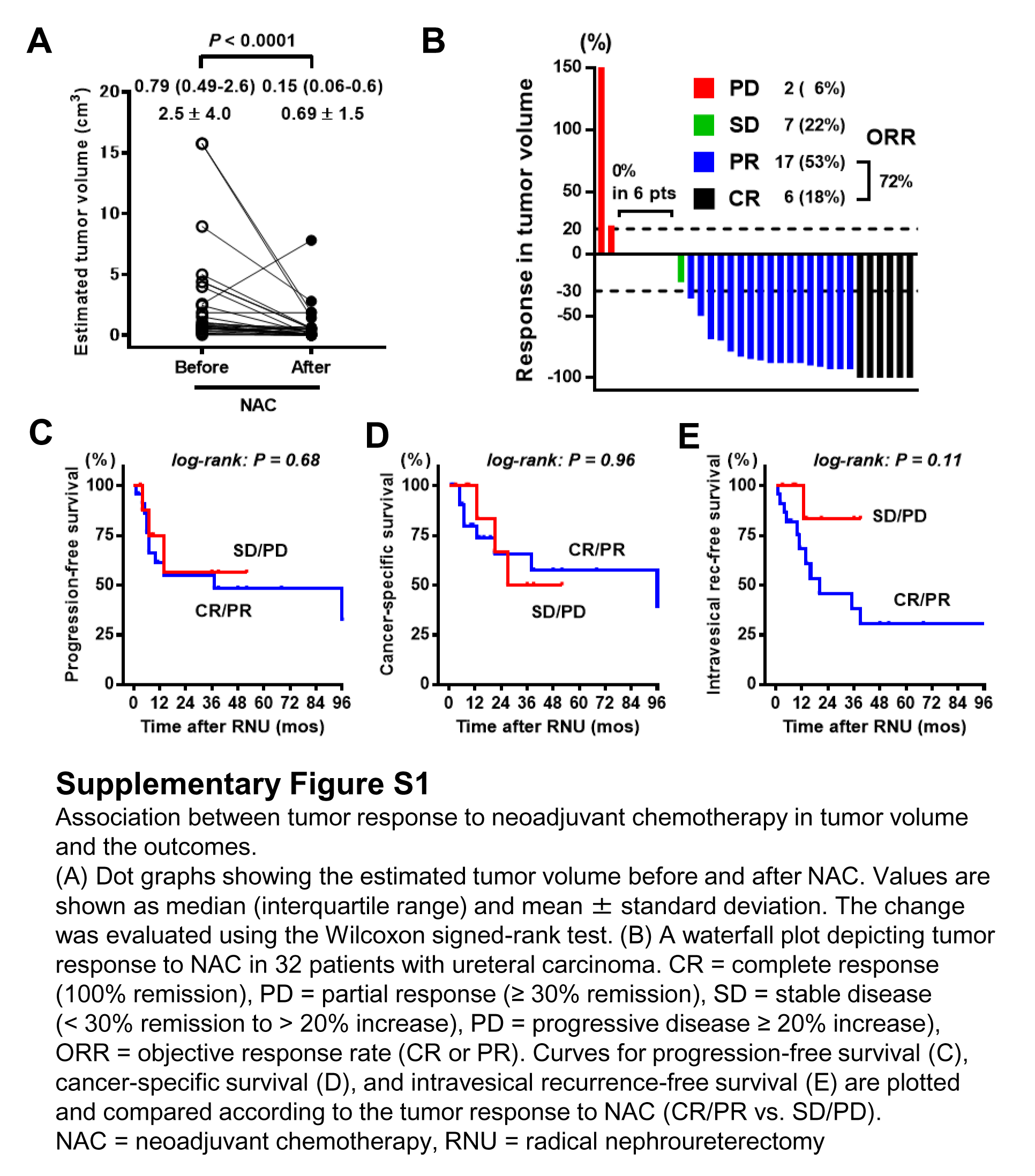

Supplement: Supplementary file 1 [file diagnostics-10-00010-s001.zip › diagnostics-648126-supplementary.tif]
